# Clinical Remarks on Dropsy

**Published:** 1884-01-20

**Authors:** G. G. Roy

**Affiliations:** Professor of Materia Medica and Lecturer on Toxicology in Southern Medical College, Atlanta, Ga.


### CLINICAL REMARKS ON DROPSY.

By G. G. Roy, M.D.,
**Professor of Materia Medica and Lecturer on Toxicology in Southern Medical College, Atlanta, Ga.**

The case I now present you is a typical one of dropsy, and belongs to a class of cases more empirically diagnosed and treated than any I can now call to mind. And why is this? The reason is obvious: Sight is lost of the fact that, pathologically considered, dropsy is not the disease per se, but the symptom and result of organic lesion, or disease.

When brought into contact with a case of this sort—too frequently—the plan of the average practitioner in dealing with it is about that of the most unmitigated dropsy quack, with which the world is now flooded. He sees the patient is swollen—it may be local or general; he pronounces, without hesitation, "It is a case of dropsy," and all we have to do "is to get rid of the water".—and this he essays to do—and sometimes succeeds—by the use of active diuretics and purgatives—and he pronounces his patient cured.

Now, gentlemen, I wish to impress this point upon you—and hope you will never forget it—that getting rid ot the water, or reducing a dropsy, *is not curing the disease.*

Another fact—of greater importance—I would impress upon you is, that when you have a case of dropsy to deal with there are three vital organs you must carefully and searchingly interrogate before you can properly diagnose and treat it.

These are the liver, heart and kidneys: in one or more of these you will find the obstruction which dams the blood from which the effusion or dropsy comes. I have mentioned them in this order because, in our malarial Southern country, the obstruction is in the liver (fortunately for the patient) more frequently than in either of the others.

I would, also, like you to remember that dropsy may be *active*, the result of acute inflammation, or *passive*, the result of chronic or continued obstruction of the veins. In *liver* dropsies you will generally find the swelling in the abdomen; in cardiac or heart dropsies,in the lower extremities, especially the feet; and in kidney dropsies, especially from albuminuria, in the eyelids and face. To an experienced observer the doughy puffiness of the eyelids and face in this last affection is almost pathogomonic.

You have a diseased liver, heart or kidneys, upon which the dropsy depends, and of which it is *a symptom.* Now, what is the rational plan of treatment for its cure? The *dropsy quacks* have learned that drastic purging of the bowels or kidneys will oftentimes reduce a patient so swollen that the integuments of the lower extremities have split or burst—and it is a shame upon the American people to say it, but it is true that this bit of knowledge is daily extending their fraudulent fame and filling their purses with ill-gotten ducats. But the quack stops here; he can go no further; he does not know whether it is a liver, a heart or a kidney dropsy, nor does he care—the sooner the dropsy re-accumulates, the sooner his purse is replenished.

But with you, gentlemen, it will not be so—it *must not* be so. Having diagnosed the cause of the dropsy—the organ diseased and its nature—you, too, by the judicious use of purgatives and diuretics, will endeavor to relieve the dropsy; having done this, you will not stop and pronounce your patient cured, but will proceed at once to remove the cause by relieving the obstruction of the veins wherever located, whether it is with the liver, the heart or the kidneys, and at the same time improve the quality of the blood.

This woman's dropsy evidently comes from obstruction of the portal circulation from a hardened liver, the result of chronic malarial poisoning, and we will place her upon Dr. Alonzo Clark's pill of

℞ Hydrg. chlo. mite.............................gr. xij,
Pulv. digitalis.............................gr. xij,
Pulv. scillæ.............................gr. xij.

M. ft. pills or capsules No. 12.

Sig. One three times daily.

And every other night a pill of

℞ El aterium (clutterbcuks).....................gr. j,
Ext. jalapaæ.............................grs. xij,
Pulv. digitalis.............................grs. xij,
Pulv. scillæ.............................grs. xij.

M. ft. pills or capsules No. 12.

You will omit the pill of the first ℞ the night this is given.

After we reduce this patient, which we may reasonably hope to do in a week's time, we should address our treatment to the hardened liver, which has obstructed the portal circulation and caused the dropsy, and for this purpose I have found nothing better than this,

℞ Hydrg. bichlorid.............................gr. j,
Ammo murias.............................ʒ iij,
Simple elixir.............................q. s. ℥ iv.

M. ft. sol.

Sig. Teaspoonful three times daily in wineglass of water.

I hope you will remember that, while using this treatment, it is all-important that you should build up the blood with the most soluble ferruginous preparation. If not, you may certainly expect the dropsy to return from the slightest imprudence of diet or temperature.

The liver of the chronic drunkard is peculiarly susceptible to the influences which produce this kind of dropsy, and in these cases I have used Prof. Robley Dunglison's ferro-saline repeatedly with most signal relief. The ferro-saline is a combination of sulphate magnesia and sulphate of iron.

